# Bezoar-Induced Small Bowel Obstruction in a Patient With a Remote History of Gastric Bypass: A Case Report

**DOI:** 10.7759/cureus.114419

**Published:** 2026-08-12

**Authors:** Jasndeep Kaler, Rickey Shah, Ulrica S Armbrister, Carisma Cherukuri, Elias Kondilis, Frederick Tiesenga

**Affiliations:** 1 Medicine, Windsor University School of Medicine, Cayon, KNA; 2 Surgery, Windsor University School of Medicine, Cayon, KNA; 3 General Surgery, Community First Medical Center, Chicago, USA; 4 General Surgery, West Suburban Medical Center, Chicago, USA

**Keywords:** bariatric surgery complication, dietary risk factors, emergency exploratory laparotomy, ileal obstruction, intraluminal mass, phytobezoar, recurrent small bowel obstruction, roux-en-y gastric bypass (rygb), small bowel enterotomy, small bowel obstruction (sbo)

## Abstract

Bezoar-induced small bowel obstruction (SBO) remains an uncommon but important consideration in patients with prior Roux-en-Y gastric bypass (RYGB), where altered gastrointestinal anatomy predisposes to impaired digestion of fibrous foods. Thus, we present a case of a 50-year-old woman with recurrent SBO who developed an acute obstruction requiring surgical intervention. Imaging suggested SBO without a clear etiology, and intraoperative exploration identified an obstructing intraluminal mass in the terminal ileum, presumed to represent a seed-containing phytobezoar based on its gross appearance and the patient’s subsequently obtained dietary history. The procedure began as a diagnostic laparoscopy and was converted to exploratory laparotomy with extraction of the obstructing intraluminal mass through an enterotomy, followed by primary closure. The involved bowel remained viable, without evidence of ischemia. This case highlights the diagnostic challenge of bezoar-related SBO, as imaging findings are often nonspecific, and emphasizes the importance of timely operative evaluation and management in appropriately selected patients. It also underscores the potential role of dietary factors, including seed consumption in this case, in bezoar formation and the need for targeted nutritional counseling in post-bariatric populations to reduce recurrence risk.

## Introduction

Small bowel obstruction (SBO) is a common cause of acute abdominal pain and accounts for a substantial number of emergency surgical admissions [1]. In patients who have undergone Roux-en-Y gastric bypass (RYGB), common causes of SBO include internal hernias, postoperative adhesions, anastomotic strictures, and intussusception [2,3]. In contrast, bezoar-induced obstruction is an uncommon etiology. A phytobezoar is a type of bezoar composed predominantly of indigestible plant material, including vegetable and fruit fibers, seeds, and other poorly digestible food components [4]. Although uncommon, phytobezoar-induced SBO is a recognized late complication following bariatric and other gastric surgery and should remain a diagnostic consideration in patients with altered gastrointestinal anatomy who present with obstructive symptoms [4,5].

RYGB produces substantial anatomical and physiological changes to the gastrointestinal tract. The procedure creates a small gastric pouch that is connected to the jejunum, thereby bypassing the majority of the stomach, the pylorus, and the proximal small intestine. These alterations affect the mechanical and chemical processing of ingested food. Reduced gastric acid exposure, loss of normal pyloric regulation, and altered gastrointestinal motility may impair the breakdown and controlled passage of fibrous or poorly digestible material, creating conditions that predispose susceptible patients to phytobezoar formation [6,7]. Seeds, in particular, contain poorly digestible plant material and have been implicated in bezoar formation in susceptible individuals [4,8].

When a phytobezoar enters the small bowel and becomes impacted, its clinical presentation may be difficult to distinguish from more common causes of postoperative SBO. Symptoms such as abdominal pain, nausea, vomiting, and obstipation are nonspecific, while imaging may establish the presence of mechanical obstruction without clearly identifying the bezoar as its underlying cause. Consequently, the etiology may not become apparent until operative exploration [4,8]. Phytobezoar-induced SBO following bariatric surgery has been previously described in case reports, case series, and systematic reviews and therefore represents a recognized, although uncommon, late complication rather than an unreported entity [5,7,8].

We report the case of a 50-year-old woman who developed recurrent SBO approximately 14 years after RYGB, with operative exploration identifying an obstructing intraluminal mass in the terminal ileum presumed to represent a seed-containing phytobezoar. The presumptive composition was supported by its gross appearance and a subsequently obtained dietary history revealing frequent consumption of a homemade trail mix containing pumpkin seeds, sunflower seeds, flaxseeds, dried fruits, and nuts. The mass was extracted through an enterotomy followed by primary closure, without segmental bowel resection. This case illustrates a recognized but uncommon late complication of RYGB while specifically highlighting the prolonged interval following bariatric surgery, a history of recurrent SBO, and a potentially modifiable dietary exposure that prompted targeted postoperative nutritional counseling.

## Case presentation

A 50-year-old woman presented to the emergency department on November 23, 2025, approximately 36 hours after symptom onset, with progressively worsening epigastric abdominal pain, nausea, and vomiting. Her symptoms began on the morning of November 22, 2025, and she reported no bowel movement or passage of flatus since symptom onset.

Her medical history was significant for recurrent SBO, paroxysmal atrial fibrillation with episodes of rapid ventricular response, gastroesophageal reflux disease, right knee osteoarthritis, pancreatitis, and seasonal allergies. Her surgical history included RYGB in 2011, laparoscopic cholecystectomy, and one prior operative intervention for SBO. She could not recall the dates of her cholecystectomy or prior SBO surgery but reported that the latter occurred several years earlier. She estimated experiencing at least five episodes of SBO during the preceding three to four years, although she was uncertain of the exact number. With the exception of the single prior operative episode, these obstructions were managed conservatively. Her home medications are summarized in Table 1.

**Table 1:** Home medications prior to admission based on patient-reported history. Dosages and frequencies reflect patient recall. Dosages and frequencies reflect patient recall.

Medication	Dose	Frequency	Indication
Apixaban	5 mg	Twice daily	Atrial fibrillation (stroke prevention)
Digoxin	0.125 mg	Once daily	Atrial fibrillation (rate control)
Diltiazem	120 mg	Once daily	Atrial fibrillation (rate control)
Pantoprazole	40 mg	Once daily	GERD
Meloxicam	15 mg	Once daily	Osteoarthritis
Ferrous Sulfate	Not specified	Daily	Supplement
Omega-3 fish oil	1000 mg	Daily	Supplement

Her family history was noncontributory. She lived independently and denied tobacco or recreational drug use. She reported occasional alcohol consumption of approximately one to three glasses of wine per week. Initial dietary history was limited; she described eating a regular diet with predominantly home-cooked meals and monitoring her caloric intake but did not initially disclose specific consumption of seeds or other high-fiber foods. A more detailed dietary history was obtained postoperatively, as described below.

On arrival at the emergency department, the patient reported progressively worsening epigastric pain associated with nausea and vomiting. She appeared uncomfortable and in mild distress but was awake, alert, and oriented to person, place, and time. Initial vital signs were within normal limits, including a blood pressure of 111/55 mmHg, heart rate of 84 beats per minute, temperature of 97.3°F (36.3°C), respiratory rate of 18 breaths per minute, oxygen saturation of 96% on room air, and a body mass index of 37.84 kg/m².

Physical examination revealed a mildly distended abdomen that was tympanic to percussion. There was tenderness to palpation localized to the epigastric region without guarding or rebound tenderness. Bowel sounds were hyperactive. No palpable masses or abdominal wall hernias were identified. Well-healed laparoscopic surgical scars were noted. A rectal examination was not performed.

Initial management included conservative measures with bowel rest, intravenous fluid resuscitation, and antiemetic therapy. The patient was made nil per os (NPO), and anticoagulation with apixaban was held in anticipation of possible surgical intervention. Although the patient presented with symptoms consistent with SBO, the obstruction was not characterized as partial or complete, and she remained clinically stable and was tolerating initial conservative management; therefore, nasogastric decompression was deferred preoperatively. An NG tube was subsequently placed intraoperatively for decompression. General surgery was consulted for further evaluation and management.

Initial laboratory evaluation on admission demonstrated marked leukocytosis with a white blood cell count of 20.3 × 10³/µL, raising concern for an acute inflammatory or obstructive process. Mild hyperglycemia (115 mg/dL) was noted, likely stress-induced in the setting of acute illness. Electrolytes were largely within normal limits, with mild hypochloremia (97 mmol/L) consistent with ongoing vomiting. Renal function remained preserved. Serum lactate and venous blood gas results were not available for assessment of possible tissue hypoperfusion. Despite the absence of these laboratory parameters, subsequent intraoperative evaluation demonstrated viable bowel without evidence of ischemia or necrosis.

Preoperative laboratory values, obtained approximately 12 hours after the initial admission studies, demonstrated a downtrend in leukocytosis to 13.1 × 10³/µL. A notable decrease in hemoglobin from 14.3 g/dL to 10.0 g/dL, hematocrit from 43.0% to 29.8%, and red blood cell count from 4.72 to 3.28 × 10⁶/µL was also observed. The etiology of this decline could not be definitively established from the available records. Potential contributing factors include hemodilution following intravenous fluid resuscitation, correction of possible hemoconcentration at presentation in the setting of vomiting and reduced oral intake, and occult blood loss, particularly given the patient’s preadmission use of apixaban and meloxicam. However, no overt source of hemorrhage was documented, and the operative report noted no active bleeding and minimal estimated blood loss. The exact volume of intravenous fluids administered between laboratory measurements and documentation regarding type and screen or a predefined transfusion threshold was not available in the records reviewed. A summary of laboratory findings on admission and prior to operative intervention is presented in Table 2.

**Table 2:** Laboratory findings on admission and preoperative. WBC: white blood cell; RBC: red blood cell; BUN: blood urea nitrogen; CO₂: carbon dioxide

Parameter	Admission (ER)	Preoperative	Reference Range	Interpretation
WBC (×10³/µL)	20.3	13.1	4.0–11.0	Leukocytosis
Hemoglobin (g/dL)	14.3	10.0	13.0–17.0	Decreased
Hematocrit (%)	43.0	29.8	38.6–49.2	Decreased
Platelets (×10³/µL)	361	263	150–450	Normal
RBC	4.72	3.28	4.34–5.60	Decreased
Glucose (mg/dL)	115	110	70–99	Likely stress-related hyperglycemia
BUN (mg/dL)	25	21	7–25	Normal
Creatinine (mg/dL)	0.67	0.69	0.60–1.30	Normal
Sodium (mmol/L)	134	135	133–144	Normal
Chloride (mmol/L)	97	96	98–109	Likely due to ongoing vomiting
Calcium (mg/dL)	9.6	9.8	8.6–10.3	Normal
CO_2_ (mEq/L)	24	23	21–31	Normal

**Figure 1:**
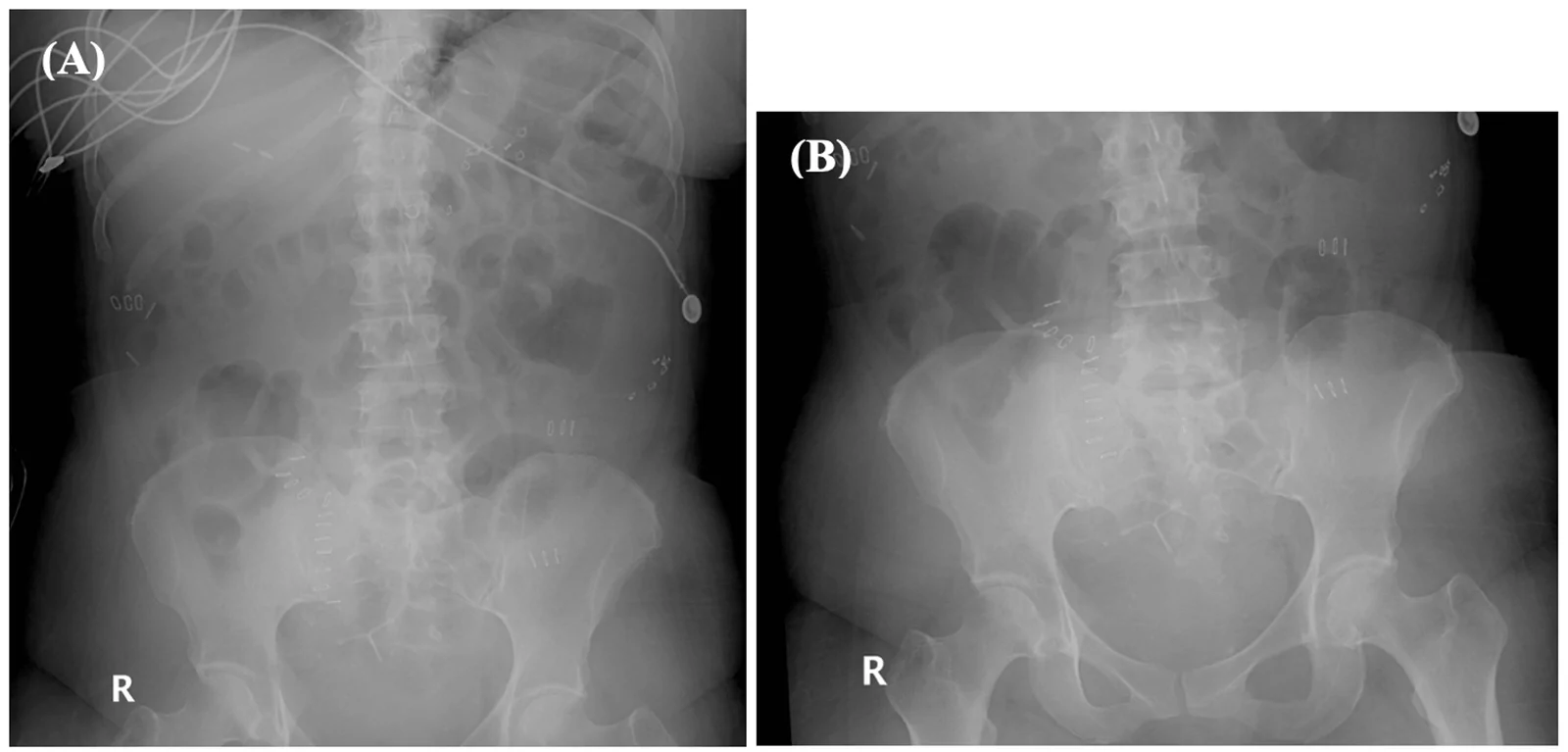
Preoperative abdominal radiographs with nonspecific findings for small bowel obstruction. (A) Supine abdominal radiograph showing nonspecific bowel gas distribution with a paucity of gas in the left colon and pelvis and no definitive radiographic evidence of small bowel obstruction. (B) Additional abdominal view demonstrating moderate stool burden throughout the colon and rectum.

As shown in Figures 1A, 1B, the abdominal radiographs demonstrated no specific findings sufficient to establish a diagnosis of SBO, although a notable paucity of bowel gas was observed in the left colon and pelvis. Moderate stool burden was also present throughout the colon and rectum. Given the limited sensitivity of plain abdominal radiography for detecting SBO, these findings did not exclude a mechanical obstruction. In the setting of persistent obstructive symptoms and continued clinical concern for SBO, further evaluation with computed tomography (CT) was pursued.

Given the patient’s history of recurrent SBO, persistent obstructive symptoms, marked leukocytosis, and continued clinical concern for mechanical obstruction despite nondiagnostic abdominal radiographs, the surgical team elected to proceed with urgent diagnostic laparoscopy, with possible conversion to exploratory laparotomy and additional bowel intervention as indicated by the intraoperative findings. Cardiology was consulted for perioperative evaluation because of the patient’s history of paroxysmal atrial fibrillation with rapid ventricular response, and she was deemed an appropriate candidate for surgical intervention. The risks, benefits, and alternatives of the procedure were discussed with the patient, and informed consent was obtained before transfer to the operating room.

**Figure 2:**
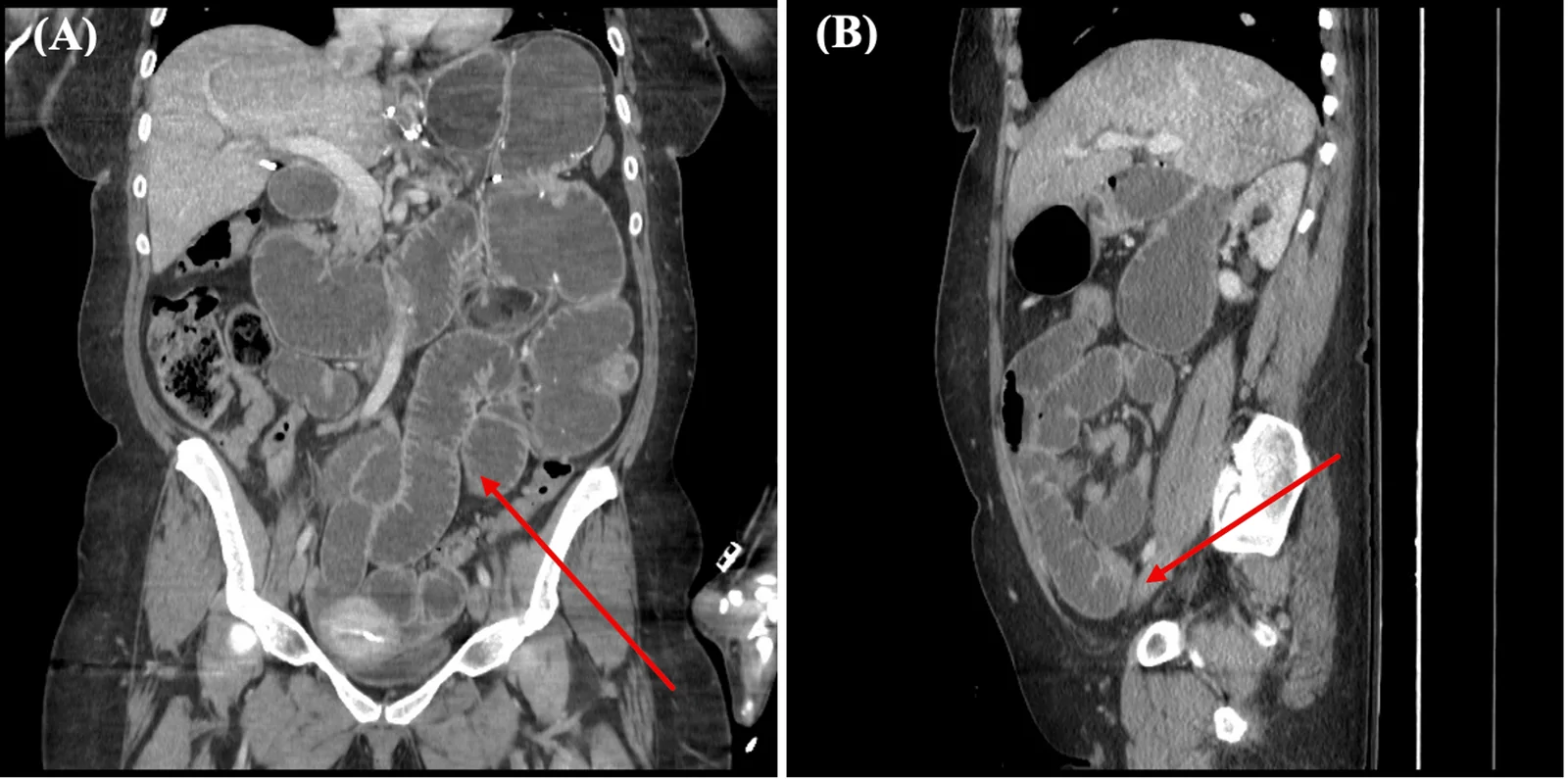
Contrast-enhanced CT of the abdomen and pelvis demonstrating findings consistent with small bowel obstruction. (A) Coronal CT image demonstrating multiple dilated, fluid-filled small bowel loops (arrow), consistent with small bowel obstruction. (B) Sagittal CT image demonstrating dilated, fluid-filled small bowel loops (arrow). A discrete transition point and the underlying etiology of the obstruction were not clearly identified on preoperative imaging.

While the patient was being prepared for surgery, the results of the CT scan of the abdomen and pelvis became available to the surgical team. The CT scan was obtained using 5-mm axial sections following administration of 90 mL of intravenous Omnipaque 350 contrast without oral contrast. As illustrated in Figures 2A, 2B, imaging demonstrated multiple dilated, fluid-filled loops of small bowel consistent with SBO. A gradual transition in bowel caliber was suggested in the mid-to-distal small bowel; however, a discrete transition point was not clearly identified, and the obstruction was not characterized as partial or complete. The CT findings supported the diagnosis of mechanical SBO and reinforced the previously made decision to proceed with operative exploration, while the precise site and etiology of the obstruction remained undetermined preoperatively.

Approximately 24 hours after presentation to the emergency department, the patient underwent operative intervention. The procedure began with diagnostic laparoscopy, which demonstrated dilated proximal small bowel loops and a transition point in the terminal ileum. The procedure was subsequently converted to exploratory laparotomy for definitive management; however, the specific reason for conversion was not documented in the available operative report. At the site of obstruction, a firm intraluminal mass measuring approximately 4-5 cm in greatest dimension and described intraoperatively as approximately the size of a golf ball was identified. Based on its gross appearance and the subsequently obtained dietary history, the mass was presumed to represent a seed-containing phytobezoar. The involved bowel appeared viable, without evidence of ischemia, necrosis, or perforation. Manual milking of the obstructing mass was not performed. An enterotomy was performed, the intraluminal mass was extracted, and the enterotomy was closed primarily. The operative report did not specify the orientation of the enterotomy, closure technique, suture material, or whether a single- or two-layer closure was performed. The extracted material and a small portion of ileal tissue excised from the enterotomy site were submitted for histopathologic evaluation. The available operative documentation did not explicitly state that the entire small bowel was systematically run; however, no additional obstructing lesions or significant intra-abdominal pathology were documented. The procedure was completed without complications, with minimal estimated blood loss and no active bleeding documented.

**Figure 3:**
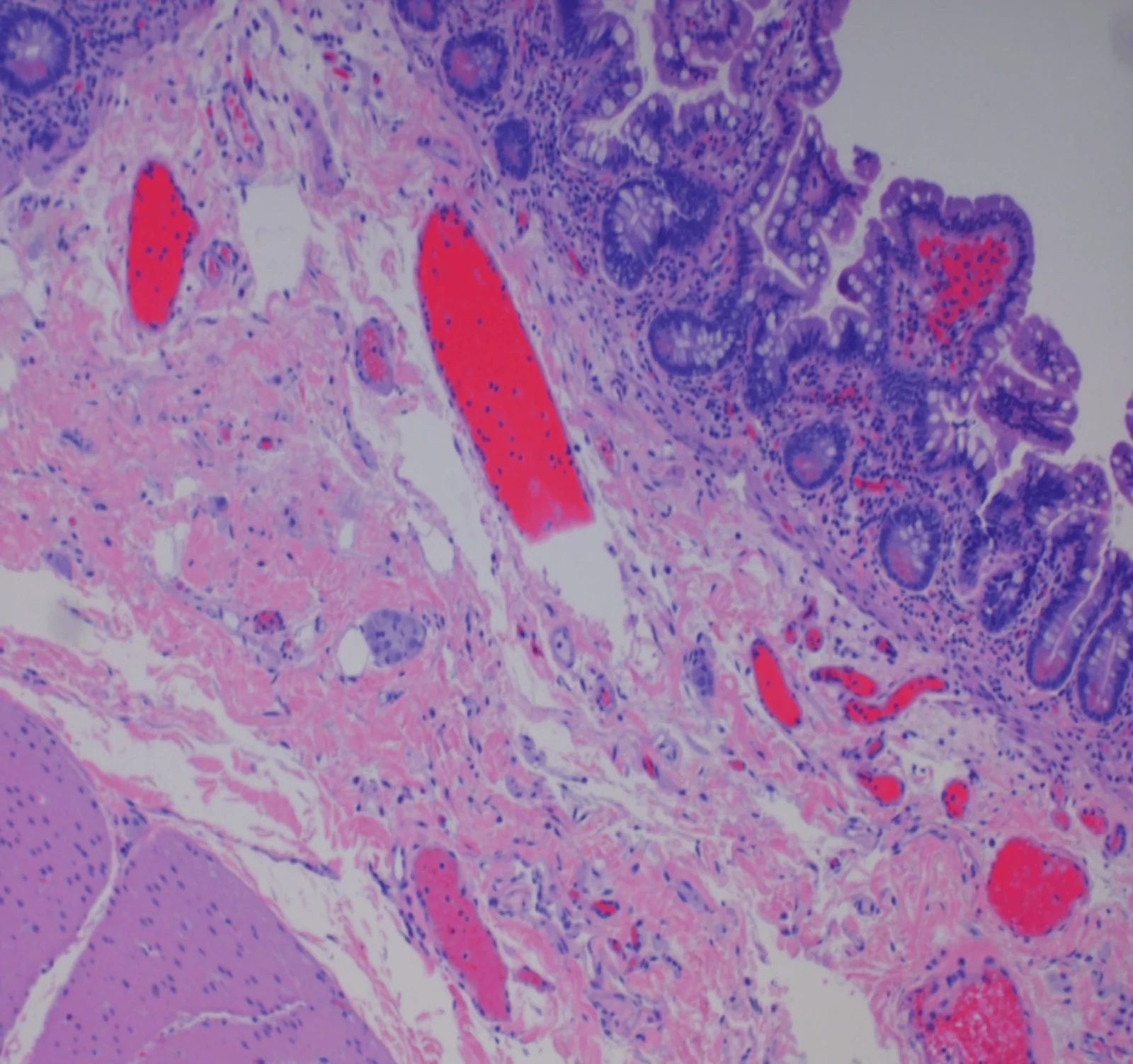
Histopathologic examination of the submitted ileal tissue demonstrating preserved mucosal architecture with focal vascular congestion and no evidence of acute inflammation, dysplasia, or malignancy.

Histopathologic evaluation of the submitted ileal tissue demonstrated small bowel mucosa with focal vascular congestion, without evidence of acute inflammation, dysplasia, or malignancy, as illustrated in Figure 3. These findings were nonspecific and did not establish the composition of the extracted intraluminal mass. The presumptive diagnosis of a seed-containing phytobezoar was therefore based on the gross operative appearance of the mass in conjunction with the subsequently obtained dietary history.

Postoperatively, the patient remained hemodynamically stable. She reported nausea without vomiting and began passing flatus. She remained alert and clinically stable without evidence of an immediate postoperative complication.

**Figure 4:**
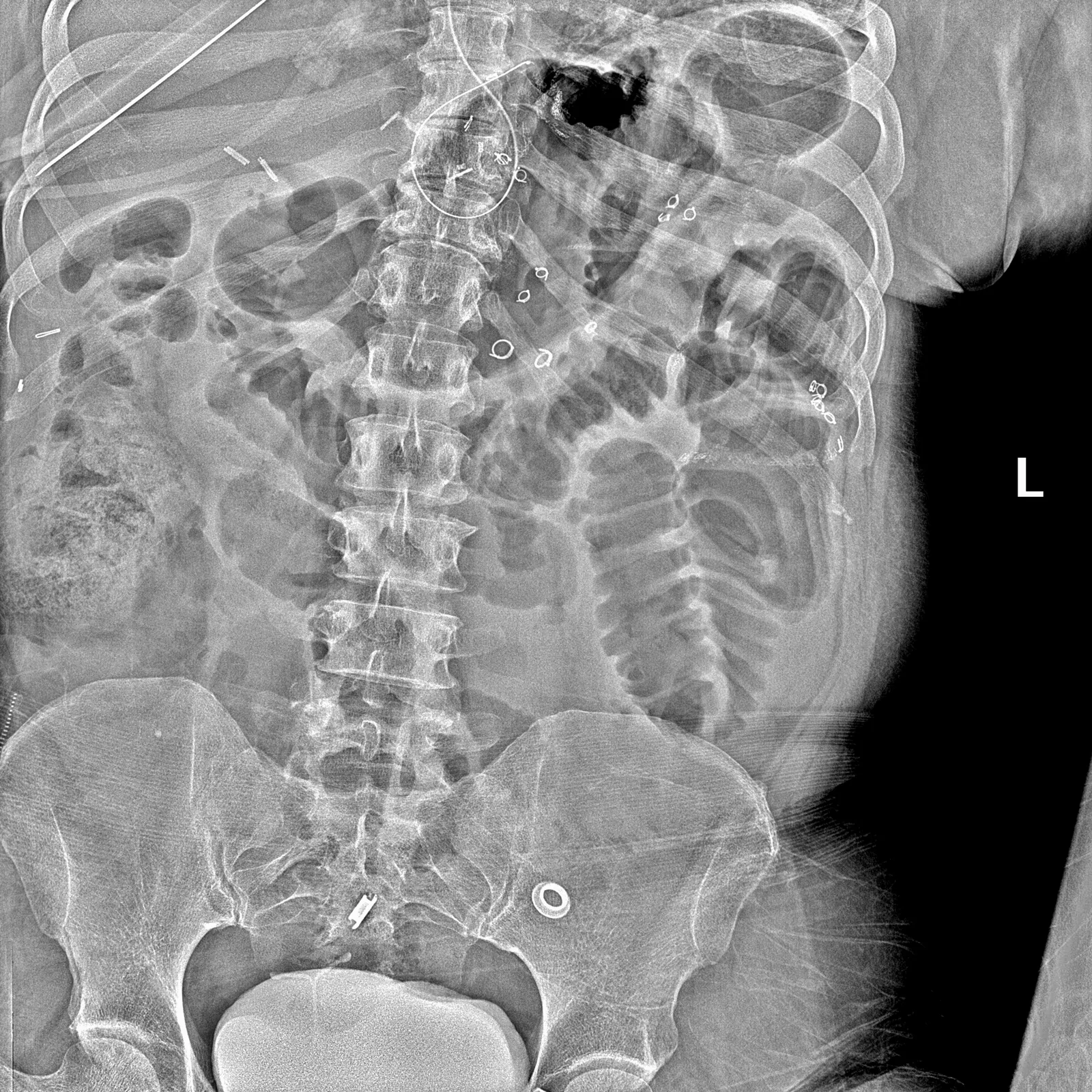
Postoperative abdominal radiograph confirming nasogastric tube placement, with the tip projecting over the gastric body.

The nasogastric tube placed intraoperatively for gastric decompression was maintained on low intermittent suction. Although output was initially minimal, the tube subsequently drained light brown fluid. Abdominal radiography confirmed appropriate positioning of the nasogastric tube tip over the gastric body, as illustrated in Figure 4. The patient was initially maintained NPO with nasogastric decompression. As gastrointestinal function returned, the nasogastric tube was removed, and oral intake was resumed and advanced as tolerated, as detailed in the postoperative course below. Early ambulation and incentive spirometry were encouraged.

Postoperatively, the patient’s leukocytosis continued to improve. Intravenous piperacillin-tazobactam and metronidazole were administered during the postoperative period. Because piperacillin-tazobactam already provides anaerobic coverage, the addition of metronidazole represented overlapping anaerobic therapy; the rationale for this combination was not documented in the available records. The patient was receiving chronic apixaban therapy for atrial fibrillation, which was held in the perioperative period. Four-factor prothrombin complex concentrate (Kcentra) was administered before surgery. However, the available records did not document the timing of the patient’s last apixaban dose or provide a specific rationale for the selection of four-factor PCC as the reversal strategy. Therapeutic anticoagulation with apixaban remained interrupted for approximately two to three days postoperatively, during which standard venous thromboembolism prophylaxis was provided. Intravenous pantoprazole was continued postoperatively, with transition to oral therapy as tolerated. A bowel regimen consisting of stool softeners, stimulant laxatives, and osmotic agents was initiated to facilitate return of bowel function. Postoperative analgesia included as-needed hydromorphone and ketorolac, with subsequent transition to oral analgesics as clinically appropriate.

The patient’s postoperative recovery was largely uncomplicated, with progressive return of gastrointestinal function. By postoperative day (POD) 2, she was passing flatus without nausea or vomiting but had not yet had a bowel movement. On POD 3, following continued clinical improvement and return of flatus, the nasogastric tube was removed, and she was advanced to a full-liquid diet, which she tolerated without recurrent nausea or vomiting. Oral intake was subsequently advanced as tolerated. On POD 5, the patient had a bowel movement, indicating return of bowel function. Her postoperative pain progressively improved and was adequately controlled with analgesic therapy. Given her return of bowel function, tolerance of oral intake, and overall clinical improvement, she was discharged in stable condition on POD 6 with outpatient follow-up with her primary care physician and surgical team.

During the postoperative course, the surgical team revisited the patient’s dietary history after discussing the intraoperative findings and considering potential contributors to her recurrent SBO in the setting of prior RYGB. At that time, the patient provided additional dietary details that had not been disclosed during the initial history. She reported frequently skipping breakfast and consuming a latte in the morning, as well as regularly snacking on a homemade trail mix containing pumpkin seeds, sunflower seeds, flaxseeds, dried fruits, and nuts, including cashews. This dietary history raised concern that frequent consumption of poorly digestible, high-fiber foods may have contributed to the formation of the presumed phytobezoar in the setting of altered gastrointestinal anatomy. The patient expressed interest in additional dietary guidance, and a nutrition consultation was arranged to reinforce recommended post-bariatric dietary practices. The key events from initial symptom onset through postoperative recovery and discharge are summarized chronologically in Table 3.

**Table 3:** Clinical timeline of presentation, diagnostic evaluation, operative management, and postoperative recovery.

Time	Clinical Event
November 22, 2025, morning	Abdominal pain, nausea, and vomiting began. The patient subsequently reported no bowel movement or passage of flatus.
November 23, 2025	The patient presented to the emergency department (ED) approximately 36 hours after symptom onset. Initial evaluation demonstrated epigastric tenderness and leukocytosis of 20.3 x 10³/µL.
Initial ED evaluation	Abdominal radiographs demonstrated nonspecific findings and did not exclude mechanical small bowel obstruction (SBO). Conservative management with bowel rest and intravenous fluid resuscitation was initiated, and apixaban was held.
Preoperative evaluation	Persistent obstructive symptoms, recurrent SBO history, leukocytosis, and continued clinical concern despite nondiagnostic abdominal radiographs prompted the decision to proceed with urgent operative exploration.
While preparing for surgery	CT results became available and demonstrated multiple dilated, fluid-filled small bowel loops consistent with SBO. A gradual transition in bowel caliber was suggested, but a discrete transition point was not clearly identified, and the obstruction was not characterized as partial or complete. The findings reinforced the previously made operative decision.
Approximately 24 hours after ED presentation	Diagnostic laparoscopy identified dilated proximal small bowel loops and a transition point in the terminal ileum. The procedure was converted to exploratory laparotomy. A 4–5 cm obstructing intraluminal mass was extracted through an enterotomy followed by primary closure. The involved bowel remained viable without evidence of ischemia or necrosis.
POD 2	The patient was passing flatus without nausea or vomiting but had not yet had a bowel movement.
POD 3	The nasogastric tube was removed, and a full-liquid diet was initiated and tolerated.
Postoperative dietary assessment	A more detailed dietary history revealed frequent consumption of homemade trail mix containing pumpkin seeds, sunflower seeds, flaxseeds, dried fruits, and nuts. A nutrition consultation was arranged.
POD 5	A bowel movement was documented.
POD 6	The patient was discharged in stable condition following return of bowel function and tolerance of oral intake.

## Discussion

SBO is a common surgical emergency, most frequently associated with postoperative adhesions [1]. In patients with prior RYGB, however, the differential diagnosis also includes internal hernias, anastomotic strictures, intussusception, and, less commonly, bezoars [2-5]. Phytobezoar-induced SBO following bariatric surgery is a recognized but uncommon late complication and has been described in case reports, case series, and systematic reviews [5,7,8]. The present case is notable for recurrent episodes of SBO occurring approximately 14 years after RYGB, with operative identification of an obstructing intraluminal mass presumed to represent a seed-containing phytobezoar.

Several anatomical and physiological changes following RYGB may predispose susceptible patients to phytobezoar formation. Reduced gastric volume, decreased exposure to gastric acid, bypass of normal pyloric regulation, and altered gastrointestinal motility may impair the breakdown and controlled passage of poorly digestible plant material [6,7]. Dietary factors may further contribute to this process. Seeds, nuts, dried fruits, and other fibrous foods have been associated with phytobezoar formation, particularly when inadequately chewed [4,5,8]. In the present case, the patient’s postoperative dietary history revealed frequent consumption of a homemade trail mix containing pumpkin seeds, sunflower seeds, flaxseeds, dried fruits, and nuts. Although a causal relationship cannot be established from a single case, this exposure represents a plausible and potentially modifiable contributor to the formation of the presumed phytobezoar in the setting of altered gastrointestinal anatomy.

The case also illustrates the diagnostic difficulty of bezoar-related SBO. Abdominal pain, nausea, vomiting, and obstipation are nonspecific and overlap with other causes of mechanical obstruction. Plain abdominal radiographs in this patient were nondiagnostic and did not exclude SBO. Subsequent CT demonstrated multiple dilated, fluid-filled small bowel loops and suggested a gradual transition in caliber in the mid-to-distal small bowel; however, a discrete transition point, the degree of obstruction as partial or complete, and the underlying etiology were not clearly established preoperatively. Although CT may demonstrate characteristic findings of a bezoar, including an intraluminal mass with a mottled gas pattern, such findings are not universally present [4,8]. In this case, the transition point in the terminal ileum and obstructing intraluminal mass were definitively localized during operative exploration.

The sequence of clinical decision-making is also relevant. The decision to proceed with operative exploration was made in the setting of persistent obstructive symptoms, marked leukocytosis, recurrent SBO, and continued clinical concern for mechanical obstruction despite nondiagnostic abdominal radiographs. The CT results became available while the patient was being prepared for surgery and supported the diagnosis of SBO, thereby reinforcing the previously made operative decision, although the precise etiology remained uncertain. Operative exploration demonstrated viable bowel without ischemia, necrosis, or perforation and permitted extraction of the obstructing mass through an enterotomy followed by primary closure. These findings support the role of timely surgical evaluation in selected patients with persistent or concerning features of SBO while avoiding the inference that operative timing itself was responsible for the absence of ischemic complications.

Several aspects of the perioperative management warrant consideration. The patient was receiving chronic apixaban therapy for atrial fibrillation, which was held perioperatively, and four-factor prothrombin complex concentrate was administered before surgery. The available records did not document the timing of the last apixaban dose or the specific rationale for selecting this reversal strategy; therefore, the appropriateness of this decision relative to alternative reversal or interruption strategies cannot be retrospectively determined from the available documentation. Similarly, postoperative piperacillin-tazobactam and metronidazole provided overlapping anaerobic coverage, but the rationale for this combination was not documented. Postoperative analgesia included hydromorphone and ketorolac. Opioid analgesics may reduce gastrointestinal motility, while nonsteroidal anti-inflammatory drugs warrant consideration of gastrointestinal and bleeding risks in the postoperative setting, particularly when therapeutic anticoagulation must subsequently be resumed. Although the patient’s first documented bowel movement occurred on POD 5, the available data do not establish that the analgesic regimen contributed to the timing of gastrointestinal recovery.

Prevention of recurrence represents an important educational aspect of this case. Patients with altered gastrointestinal anatomy following bariatric surgery may remain susceptible to bezoar formation years after the original procedure [5,7]. Dietary counseling regarding appropriate food selection, adequate chewing, hydration, and adherence to post-bariatric dietary recommendations may therefore be important, particularly in patients with recurrent obstructive episodes [5,6]. In this case, the more detailed postoperative dietary assessment identified frequent consumption of seeds and other fibrous foods that had not been disclosed during the initial history. This finding prompted a nutrition consultation and targeted dietary counseling. The case therefore reinforces the value of obtaining a detailed dietary history in post-bariatric patients presenting with recurrent or otherwise unexplained SBO.

The prolonged interval between RYGB and the present obstruction further demonstrates that bezoar-related complications may occur remotely after bariatric surgery. Clinicians evaluating patients with previous RYGB should therefore maintain a broad differential diagnosis rather than attributing recurrent obstruction solely to more prevalent postoperative causes such as adhesions or internal hernias. The existing literature, including reports by Wang et al. [9] and Caputo et al. [10], similarly describes the diagnostic and therapeutic challenges associated with bezoar-induced SBO and supports consideration of bezoars in patients with altered gastrointestinal anatomy.

The unusual etiology in this case is further highlighted when considered alongside the more prevalent causes of SBO following RYGB. Hwang et al. reported SBO in approximately 3% of patients following laparoscopic gastric bypass, with common etiologies including adhesions, obstruction related to the jejunojejunostomy, and internal hernias [2]. Similarly, Elms et al., in a review of 2,395 RYGB cases, found that adhesions and internal hernias accounted for a substantial proportion of reoperations for suspected SBO, with intussusception representing another recognized cause [3]. These findings illustrate that postoperative SBO following RYGB is more commonly associated with adhesive, anatomic, or internal hernia-related mechanisms, supporting the importance of maintaining less common intraluminal etiologies, including bezoars, in the differential diagnosis when more typical causes are not identified [2,3].

Limitations

This report has several limitations. As a single case, its findings cannot establish causality or be generalized to all patients with prior RYGB. The composition of the extracted intraluminal mass was not chemically or morphologically confirmed, and histopathologic examination of the submitted ileal tissue demonstrated nonspecific focal vascular congestion without findings that established the composition of the mass. Accordingly, the diagnosis of a seed-containing phytobezoar remains presumptive and is based on the gross operative appearance together with the subsequently obtained dietary history. Preoperative imaging demonstrated SBO but did not clearly identify the intraluminal mass or a discrete transition point. In addition, portions of the retrospective clinical documentation were incomplete, including the rationale for conversion from laparoscopy to laparotomy, whether the entire small bowel was systematically examined for synchronous bezoars, details of the enterotomy closure technique, and the rationale for selected perioperative medication decisions. These limitations restrict retrospective assessment of some aspects of the patient’s management.

## Conclusions

This case highlights bezoar-induced SBO as an uncommon but important consideration in patients with a history of RYGB. In this patient, CT confirmed SBO but did not clearly identify a discrete transition point or establish the underlying etiology. Operative exploration subsequently localized the transition point to the terminal ileum and identified an obstructing intraluminal mass presumed to represent a seed-containing phytobezoar based on its gross appearance and the subsequently obtained dietary history. The mass was extracted through an enterotomy followed by primary closure, with preservation of viable bowel and no need for segmental bowel resection. This case emphasizes the importance of considering bezoars in the differential diagnosis of recurrent or unexplained SBO following RYGB, particularly when imaging confirms obstruction without clearly identifying its cause. The patient’s frequent consumption of seeds and other fibrous foods represents a potentially modifiable dietary factor that may have contributed to phytobezoar formation. Detailed dietary assessment and targeted nutritional counseling may therefore be valuable components of management and recurrence prevention in susceptible post-bariatric patients.
